# Opioid-induced Somatic Activation: Prevalence in a Population of Patients With Chronic Pain

**DOI:** 10.7759/cureus.7911

**Published:** 2020-05-01

**Authors:** Andrew Barbour, Melinda L Asbury, Paul A Riordan, Jason A Webb, Steven D Prakken

**Affiliations:** 1 Anesthesia, Duke University School of Medicine, Durham, USA; 2 Psychiatry, University of North Carolina at Chapel Hill School of Medicine, Chapel Hill, USA; 3 Psychiatry and Behavioral Sciences, Duke University School of Medicine, Durham, USA; 4 Psychiatry, Durham Veterans Affairs Medical Center, Durham, USA; 5 Palliative Care, Duke University Health System, Durham, USA; 6 Psychiatry, Duke University Health System, Durham, USA

**Keywords:** opioid, opiate, sleep disturbance, energy, depression

## Abstract

Context and objective

Opioids have heterogeneous side effects including a well-known effect of sedation; however, the opposing effect of stimulation, or somatic activation, has been largely ignored or overlooked. The objective of this study is to determine the prevalence of opioid-induced somatic activation (OISA).

Methods

We conducted a retrospective chart review of 189 patients seen by a single clinical psychiatrist/pain specialist. During the initial encounter, the clinician took a standardized history of every opioid currently or previously taken by the patients, and enquired if the patients had experienced a somatically activating or sedating effect per opioid.

Results

Patients recalled an average exposure to 5.1 opioids (SD: 1.9). Ninety-one patients (48.1%; mean: 1.6) reported somatic activation, while 118 (62.4%; mean: 1.7) reported sedation from at least one opioid. Fifty-eight patients (30.7%) identified at least one opioid as activating, and another as sedating. The distribution of OISA did not significantly differ by gender, race, primary pain diagnosis, or depression. The distribution of OISA by oxycodone significantly differed compared to morphine sulfate (27.3% vs 8.9%; p: 0.005), while sedation did not (29.0% vs 24.3%; p: 0.46).

Conclusions

In this study, we quantified the previously unstudied phenomenon of OISA. This phenomenon appears dependent on opioid type with some opioids, such as oxycodone, appearing more likely to have this effect. Given current concerns about the risks of opioids in high-risk populations, future studies are needed to study this phenomenon to arrive at an accurate determination of the potential risks and benefits of OISA.

## Introduction

In 2016, 50 million adults in the US were reported to be affected by chronic pain, a debilitating and complex medical condition that is increasingly difficult to manage during an opioid-use disorder and overdose crisis [1,2]. According to the 2015 National Survey on Drug Use and Health, 91.8 million US civilian adults used prescription opioids; 11.5 million misused them at least once during the year, and 1.9 million had an opioid-use disorder [3]. The most commonly reported reason for misusing opioids was relief from physical pain, underscoring the difficulty of successfully managing chronic pain with opioids [3]. Regulatory measures that have reduced the quantity of prescribed opioids have also had potentially negative consequences such as reductions in opioid prescriptions by oncologists for cancer-related pain [4,5]. In an era of increasing opioid stigmatization and regulation, an improved understanding of how opioids affect the subjective patient experience is needed so that healthcare providers can appropriately prescribe opioids to improve a patient’s function.

Opioids have a heterogeneous set of effects, with the individual patient and pharmacologic characteristics influencing the patient experience. For example, individuals carry different genetic predispositions for opioid-use disorder, and genetic polymorphisms can affect the pain experience [6,7]. Additionally, the affective state may relate to dynamic changes in µ-opioid neurotransmission, meaning mood may affect the opioid experience at a biochemical level [8]. This may explain the increased risk of opioid abuse and dependence [odds ratio (OR): 1.46] in patients with mental health disorders, and increased risk of overdose with depression [9,10]. Finally, different opioids used in the same patient can lead to different effects due to both the variable affinity and selectivity for, and the differential activation of, opioid receptors [11-13]. Insights from heterogeneous effects have been leveraged for developing new pharmaceuticals and improving existing prescribing practices.

Sedation is a well-known opioid effect; however, the opposing effect of stimulation has been largely ignored or overlooked [14]. In our clinical experience with patients in chronic pain, we have encountered a substantial population reporting response to opioids characterized by traits such as sleep disturbance, cognitive stimulation, increased energy, and increased functional capacity. We term this response opioid-induced somatic activation (OISA). OISA is a cause for concern in chronic pain patients since anxiety and sleep disturbances are common comorbidities [15,16]. OISA may increase the patient's desire to use sedative medications like benzodiazepines, which act synergistically with opioids as a respiratory depressant and increase overdose risk four-fold [17-19]. During this era of the opioid crisis, an understanding of this activating effect is required.

The purpose of this study is to quantify the proportion of patients with chronic pain who have had the subjective experience of OISA and determine if the likelihood of OISA varied with patient factors or opioid class. Through this investigation, we aim to inform clinical decision-making about prescribing opioids to reduce a patient’s illness burden. We also hope that our findings can guide future studies on this topic.

## Materials and methods

We conducted a retrospective chart review of new patients seen by a single clinical psychiatrist and pain specialist (author S.D.P.) at the Medical Pain Service (MPS) of the Duke Pain Medicine outpatient clinic in Durham, NC from January 2014 through December 2015. The MPS specializes in the treatment of complex pain patients with psychiatric comorbidities. This research was approved by the Duke University Institutional Review Board, permitting analysis of electronic health records with a waiver of consent (Pro00102929). The chart review focused solely on the medical notes of the initial patient encounter. We extracted information including age, sex at birth, primary pain diagnosis, a current or prior diagnosis of major depressive disorder (MDD), and opioid-use history. Primary pain diagnoses were categorized as follows: cervical-spinal or lumbar-spinal pain, sickle cell disease, fibromyalgia, complex regional pain syndrome (CRPS), and other (e.g., chronic pancreatitis, headache, rheumatologic disease, multiple sclerosis, gunshot wounds, and musculoskeletal pain not including a primary cervical or lumbar etiology). Due to the patient complexity at the MPS, only the primary pain diagnosis was recorded, even when multiple pain-related comorbidities existed.

The standardized opioid history included each unique opioid currently or previously taken. For each opioid, the clinician asked if the patient recalled experiencing an activating or sedating effect. The verbal questions generally included: “Does this medication make you tired? Does this medication give you energy or wake you up at all?” When a patient was equivocal, follow-up questions included: “Do you get up and do more an hour after taking it? Do you talk more? Does your mind move faster? Does it bother your sleep? Do you take it to get going in the morning?” When reporting enhanced function, patients were asked to clarify if they were more functional due to pain relief or a perceived change in energy level. If a patient reported that they could do more due to a lower pain level, described the opioid’s effect as euphoric, or could not differentiate feelings of somatic activation from euphoria, they were not classified as being somatically activated. When conducting chart review, we coded the response for each opioid as follows: sedating, somatically activating, neutral, equivocal, or unknown. For analysis, we collapsed these codes to the following: sedating, somatically activating, or neither (combining neutral, equivocal, and unknown).

We performed statistical analyses in R statistical software version 3.5.1 (R Foundation for Statistical Computing, Vienna, Austria) [20]. Comparisons were conducted to determine if gender, race, primary pain diagnosis, or specific opioid were associated with binary variables for activation (yes/no) or sedation (yes/no). A comparison was then conducted for oxycodone and morphine sulfate. We defined significance at a p-value of <0.05 level and used the Pearson χ2 test with Yates’ continuity correction or, when expected values were low, Fisher’s exact test with p-values computed via Monte-Carlo simulations (n = 2,000).

## Results

The 189 patients included 85 males and 104 females; the average age was 46.1 years (Table 1). The majority were non-Hispanic white (Table 1). The distribution of somatic activation (p: 0.94) and sedation (p: 0.67) did not significantly differ by sex. There was no significant difference in the distribution of activation (p: 0.82) or sedation (p: 0.61) by race. The distribution of activation (p: 0.75) and sedation (p: 0.09) did not significantly differ among primary-pain diagnoses.

**Table 1:** Demographic characteristics of the study cohort SD: standard deviation; CRPS: complex regional pain syndrome

Characteristics	Male (n = 85)	Female (n = 104)
Age, years, mean (SD)	46.1 (15.1)	46.1 (14.1)
Race, n		
Non-Hispanic white	58	79
Non-Hispanic black	25	25
Other	2	0
Primary pain diagnosis, n		
Cervical or lumbar pain	40	27
CRPS	2	7
Fibromyalgia	5	24
Sickle cell disease	17	13
Other	21	33

There were 100 patients with a diagnosis of MDD (53%), and the distribution of activation (p: 0.33) and sedation (p: 0.56) did not significantly differ by MDD status. At the initial encounter, 138 patients (73%) had a documented psychiatric comorbidity of MDD, bipolar, attention deficit hyperactivity, borderline personality, post-traumatic stress, or generalized anxiety disorders. Patients recalled a mean exposure to 5.1 opioids (SD: 1.9). Ninety-one patients (48.1%) had a patient-reported outcome of somatic activation, identifying 1.6 activating opioids on average. In contrast, 118 patients (62.4%) reported at least one sedating opioid, identifying 1.7 sedating opioids on average. Additionally, 58 patients (30.7%) identified at least one opioid as activating, while identifying another as sedating.

Exposure to at least one oxycodone formulation was documented for 176 patients, with a mean exposure to 1.4 formulations. Of the 176 oxycodone-experienced patients, 48 (27.3%) reported somatic activation and 51 (29.0%) reported sedation. This included seven (4%) reporting activation and sedation depending upon oxycodone formulation. In contrast, 123 patients had exposure to at least one formulation of morphine sulfate with a mean exposure to 1.1 formulations. Of these 123, 11 (8.9%) reported activation, 30 (24.3%) sedation, and none reported variable activation and sedation. The distribution of somatic activation by oxycodone compared to morphine significantly differed (p: 0.005), while sedation did not (p: 0.46).

## Discussion

We reported on a previously unquantified observation of OISA. Nearly half of our cohort recalled experiencing somatic activation while taking an opioid, and the distribution of activation did not significantly differ by gender, race, primary pain diagnosis, or depression. While placebo effects are common in pain medicine, the statistically significant three-fold difference in activation by oxycodone vs. morphine sulfate, contrasted with equivalent rates of sedation, suggests that this is not a placebo effect or sampling artifact [21].

Clinical applications

Identifying and understanding OISA is clinically relevant due to potential patient harm from sleep disturbance and hyperactive delirium, and potential benefit from increased functional capacity. OISA can include sleep disturbance, which is concerning due to the high incidence of comorbid sleep disorders among patients with chronic pain and the finding that decreased sleep can increase pain [15,22]. Further, sleep disturbance may increase polypharmacy, including the use of benzodiazepines, thereby increasing the risk of overdose and behavioral disturbances such as delirium [17-19,23,24]. In contrast to this potential harm, somatic activation can be leveraged for patient benefit when functional capacity is low. This benefit is described by patient interviews by Back et al. in 2011: “one participant stated that prescription opioids help her ‘get started, like coffee’ in the morning… another participant stated, ‘it seems like they energize me, give me a lot more energy and seems like they sharpen my thought process’” [14]. This is particularly relevant in the palliative care setting with significant comorbidities of fatigue.

These findings may be directly translated to improve clinical care and opioid prescribing practices. We believe that eliciting a patient’s past experiences with opioid-induced energy changes prior to prescribing an opioid, and again on follow-up, should become standard practice. This baseline history can inform treatment decisions, such as transitioning patients with sleep disturbance from oxycodone to morphine formulations when appropriate, or changing opioid formulation if a patient’s OISA manifests with irritability. When a patient has gained functional capacity via somatic activation, caution should be taken when changing opioid dose or formulation since changes in somatic activation can impact functional assessment outside of the patient’s pain report. Further, dose reduction can be difficult in patients experiencing somatic activation due to loss of stimulation, which can be mitigated by replacing this effect with another agent (e.g., bupropion). Such an approach can make opioid reduction easier in an era of tightened prescribing practices [25,26].

Limitations and future directions

This study was limited by being a retrospective chart review of patient-reported outcomes documented by a single clinician without a standardized assessment tool. Data interpretation may have been confounded by multiple formulations of an opioid being prescribed to a given individual, as opposed to standardizing treatments with generic oxycodone, generic morphine sulfate, etc. Additionally, no data existed to identify the presence of dose-dependent effects. Finally, while we believe the mental health comorbidities of this cohort are generally representative of real-world patients, the naturalistic nature of this study complicates interpretation.

The next step will include delineating clear criteria for OISA so that assessment tools incorporating somatic activation can be developed and validated. Assessment tools will then allow investigations of the relationship between somatic activation and analgesia, i.e., whether suppression of pain leads to a subjective feeling of energy or, conversely, whether somatic activation affects the pain experience. When conducting this analysis, it will be important to determine if the direction of this relationship is homogenous or heterogeneous among patients. From this understanding, drivers of activation can be investigated. For example, catechol-O-methyltransferase single nucleotide polymorphisms known to be protective against sedation among patients taking morphine, while appearing insensitive to oxycodone, can be studied in relation to the differential activation and sedation documented here [27,28]. Finally, it should be elucidated if somatic activation increases the risk of opioid-use disorder. This is vital to improving prescribing practices that put patients at risk or, alternatively, to ensure somatically-activated patients are not harmed by clinical misinterpretation of their opioid-use disorder risk.

## Conclusions

The statistically significant three-fold difference in activation by oxycodone vs. morphine sulfate, contrasted with equivalent rates of sedation, suggests that the patient-reported OISA is not a placebo effect or sampling artifact. The results of this study can be used for improving clinical care practices, such as transitioning patients with sleep disturbance from oxycodone to morphine formulations when appropriate, or changing opioid formulation if a patient’s OISA manifests with irritability. Prospective data are needed to further define and quantify OISA, due to its clinical relevance relating to potential patient harm from sleep disturbance and delirium, and potential benefit from increased functional capacity.
